# Gastrointestinal endoscopy for intestinal dysplasia and neoplasia detection and management in Crohn’s disease: when and how?

**DOI:** 10.3389/fgstr.2025.1626610

**Published:** 2025-07-25

**Authors:** Tommaso Pessarelli, Alessandra Piagnani, Gian Eugenio Tontini, Irene Maria Bambina Bergna, Arnaldo Amato

**Affiliations:** ^1^ Digestive Endoscopy and Gastroenterology Department, Ospedale A. Manzoni, Lecco, Italy; ^2^ Department of Pathophysiology and Transplantation, University of Milan, Milan, Italy; ^3^ Gastroenterology and Endoscopy Unit, Fondazione IRCCS Ca’ Granda Ospedale Maggiore Policlinico, Milan, Italy

**Keywords:** endoscopy and Crohn’s disease, colorectal neoplasia and Crohn’s disease, small bowel neoplasia and Crohn’s disease, cancer and Crohn’s disease, dysplasia and Crohn’s disease

## Abstract

Patients with long-standing inflammatory bowel disease (IBD) involving the colon have an approximately 2–3-fold risk of colorectal cancer (CRC), which remains a leading cause of mortality in this population. However, data specifically assessing CRC incidence in Crohn’s disease (CD) are limited, and these patients also face an increased risk of small bowel cancer (SBC). Endoscopy plays a central role in CRC prevention, as well as in the detection and management of dysplasia and early CRC in CD. This review summarizes current evidence on the role of lower gastrointestinal endoscopy, as well as small bowel capsule endoscopy and device assisted enteroscopy, in this context. It provides practical guidance on the optimal use of these endoscopic techniques, considering patient- and disease-specific factors. Additionally, it highlights emerging endoscopic technologies and future perspectives in the field.

## 1 Introduction

Patients with inflammatory bowel disease (IBD) are at increased risk of developing gastrointestinal (GI) cancers (1). While this risk is well documented in ulcerative colitis (UC), in Crohn’s disease (CD) it is considerably more heterogeneous—both in terms of incidence and cancer location—depending on specific disease characteristics (2). Notably, most current evidence on cancer prevention, surveillance, and management in IBD is derived from UC-predominant cohorts (3). As a result, existing guidelines often fail to adequately differentiate between UC and CD in terms of cancer risk profiles and preventive strategies (2). Additionally, patients with CD are at increased risk of other GI malignancies, including small bowel cancer (4), an area with limited scientific evidence, as well as pouch and anal cancers, although the latter two are beyond the scope of this review. This scarcity of data hampers the development of specific, evidence-based recommendations for prevention and clinical management. In this context, GI endoscopy—supported by recent technological advances—is expected to play a central role in managing these patients. However, given that these techniques are often invasive and costly, their use must be carefully tailored to individual patient characteristics and risk profiles. This review summarizes current scientific evidence on the epidemiology and risk factors for GI cancers in CD and discusses recent technological advancements in endoscopy alongside future perspectives.

## 2 Crohn’s disease and colorectal cancer

### 2.1 Epidemiology and risk factors

Although some studies did not observe an increased risk of colorectal cancer (CRC) in CD (5–8), likely due to limited sample sizes and statistical power, two meta-analyses involving 6 and 34 studies, respectively, reported a 1.9- to 2.4-fold increased risk (9, 10). A declining trend has been observed in more recent years, potentially due to longer follow-up periods enabling higher dysplasia detection rates—as seen in UC (11)—and the more frequent achievement of deep remission through biologic therapies (12). Nevertheless, CRC risk in CD remains highly heterogeneous and is influenced by both disease- and patient-specific factors (2). Notably, CD without colonic involvement does not confer increased CRC risk, and thus, surveillance colonoscopy is not indicated for isolated small bowel disease (13–15). In a Hungarian longitudinal study, the stenosing phenotype was most strongly associated with CRC risk (5%) (16). Patient-related risk factors include male sex and early age at disease onset (17). A large retrospective Scandinavian cohort study reported a relative risk of 2.6 in males versus 1.9 in females (17). In contrast, the association between high inflammatory burden and increased neoplasia risk appears more relevant in UC (18). For example, the association between pseudo-polyps (as a marker of previous severe inflammation) and CRC risk was not confirmed in studies that also included CD patients (19, 20). While untreated dysplasia is expected to increase malignancy risk, most supporting data originate from UC studies (11). Patients with perianal disease also appear to have elevated CRC risk; 37% of CD patients with rectal cancer had perianal involvement (21). Although primary sclerosing cholangitis (PSC) is associated with increased CRC risk in CD, the association is weaker than in UC. In a large UK retrospective study of 2,588 IBD patients with PSC, the link reached only borderline statistical significance (22).

### 2.2 Endoscopy for detection of colorectal dysplasia and neoplasia: from high-definition white light endoscopy and chromoendoscopy to future perspectives

High-quality colonoscopy is essential for CRC and dysplasia screening or surveillance in CD. Adequate bowel cleanliness is a non-negotiable prerequisite (23, 24). Patients with IBD may use either high- or low-volume polyethylene glycol (PEG), preferably in split regimens, which offer comparable efficacy (25). Oral sodium phosphate should be avoided as it may cause aphthous ulcers, potentially confounding inflammation assessment (26). Minimal or absent mucosal inflammation is another essential factor for high-quality surveillance, although often difficult to achieve in clinical practice (27), especially in patients with refractory disease.

High-definition white light endoscopy (HD-WLE) offers better visualization of mucosal and glandular detail than standard-definition WLE and should be routinely available in IBD referral centers (28, 29). Chromoendoscopy enhances mucosal contrast and improves the visualization of superficial patterns and the vascular network. Dye-Based Chromoendoscopy (DCE) requires the application of staining agents, while virtual electronic chromoendoscopy (VEC) uses digital and optical filtering without dyes (30). Comparative data on these modalities in CD are lacking, as most studies focus on UC. A multicenter randomized controlled trial including both UC and CD patients found no difference in neoplasia detection between HD-WLE and VEC (i-Scan) (31). Other studies also found no differences among DCE, VEC, and HD-WLE for dysplasia detection in IBD (32, 33). A retrospective study on long-standing ileocolonic CD found similar dysplasia detection rates with VEC and DCE, although VEC significantly reduced withdrawal time (34). A recent meta-analysis of randomized trials (n=2,514) confirmed DCE’s superiority over SD-WLE, but found no statistical advantage over VEC. Notably, only a small portion of patients in the meta-analysis had CD, limiting the generalizability of findings (35) (**Table 1**). Nevertheless, many studies lack subgroup analyses for CD, likely due to small sample sizes (31, 36–38) (**Table 1**). Procedure duration is also a relevant consideration, with DCE significantly increasing exam time (39).

**Table 1:** Randomized controlled trials comparing endoscopic techniques for colorectal neoplasia surveillance in IBD, including patients with Crohn’s disease.

First Author & year	Study country	Endoscopic surveillance techniques	Biopsy protocol	Study population, N	CD patients included, n	Neoplastic lesions, n	Neoplastic lesions in CD, n	Subgroup analysis UC vs CD	Withdrawal time
Kandiah (30), 2021	UK	i-scan HD-WLE	Random + targeted	188	57	52	NA	NA	25.5 ± 1.7 vs 24.0 ± 1.7
Gonzalez-Bernardo (76), 2021	Spain	DCE vs i-scan	Targeted	129	25	19	4	NA	14 ± 0.8 vs 10 ± 1.8
Alexandersson (37), 2020	Sweden	DCE vs HD-WLE	Random + targeted	305	116	31	NA	NA	24 ± 11 vs 17 ± 8
Iacucci (77), 2018	Canada	HD-WLE vs DCE vs i-scan	Targeted	270	136	92	NA	Not different neoplasia detection rate	15.4 ± 2.0 vs 16.2 ± 2.2 vs 15.3 ± 2.8
Pellisé (78), 2011	Spain	NBI vs DCE (tandem)	Targeted	60	10	10 with NBI and 12 with DCE	NA	NA	15.7 ± 5.6 vs 26.9 ± 9.9

HD-WLE, High Definition White Light Endoscopy; DCE, Dye-based chromoendoscopy; NBI, Narrow Band Imaging; NA, data not available at full-text screening; CD, Crohn’s disease; UC, Ulcerative Colitis.

Both HD-WLE and chromoendoscopy are useful for guiding biopsies. Although most studies focus on UC, some indicate that targeted biopsies using either HD-WLE or chromoendoscopy can be as effective as random biopsies in detecting dysplasia (40). However, a large prospective study of 1,000 IBD patients found that approximately 15% of neoplasia cases were detected via random biopsies after DCE (41). Thus, latest British Society of Gastroenterology guidelines suggested additional quadrantic mapping non-targeted biopsies every 10 cm or from each colonic segment for specific patient risk groups, including patients with PSC, a history of colonic dysplasia in the past 5 years and patients undergoing segmental colectomy (37).

Artificial intelligence (AI) shows promise in enhancing dysplasia detection, although current literature on its use in IBD patients is scanty, including only two original studies and one systematic review (42). Indeed, current AI devices for computer-aided detection and diagnosis perform worse in IBD settings, mainly because patients with IBD were specifically excluded from the datasets used to train these algorithms (37).

Ultra-magnification techniques like probe-based confocal laser endomicroscopy (pCLE) and endocytoscopy allow for real-time or cellular-level imaging of colonic mucosa. A meta-analysis of nine studies using pCLE for *in-vivo* characterization of colonic lesions in IBD reported pooled sensitivity of 87%, specificity of 94%, and AUROC of 0.96 (43). However, the use of these modalities for neoplasia detection and characterization in IBD remains currently limited to research settings due to costs, procedural time, and required training (44).

### 2.3 Endoscopy for management of colorectal dysplasia and neoplasia

**Figure 1:**
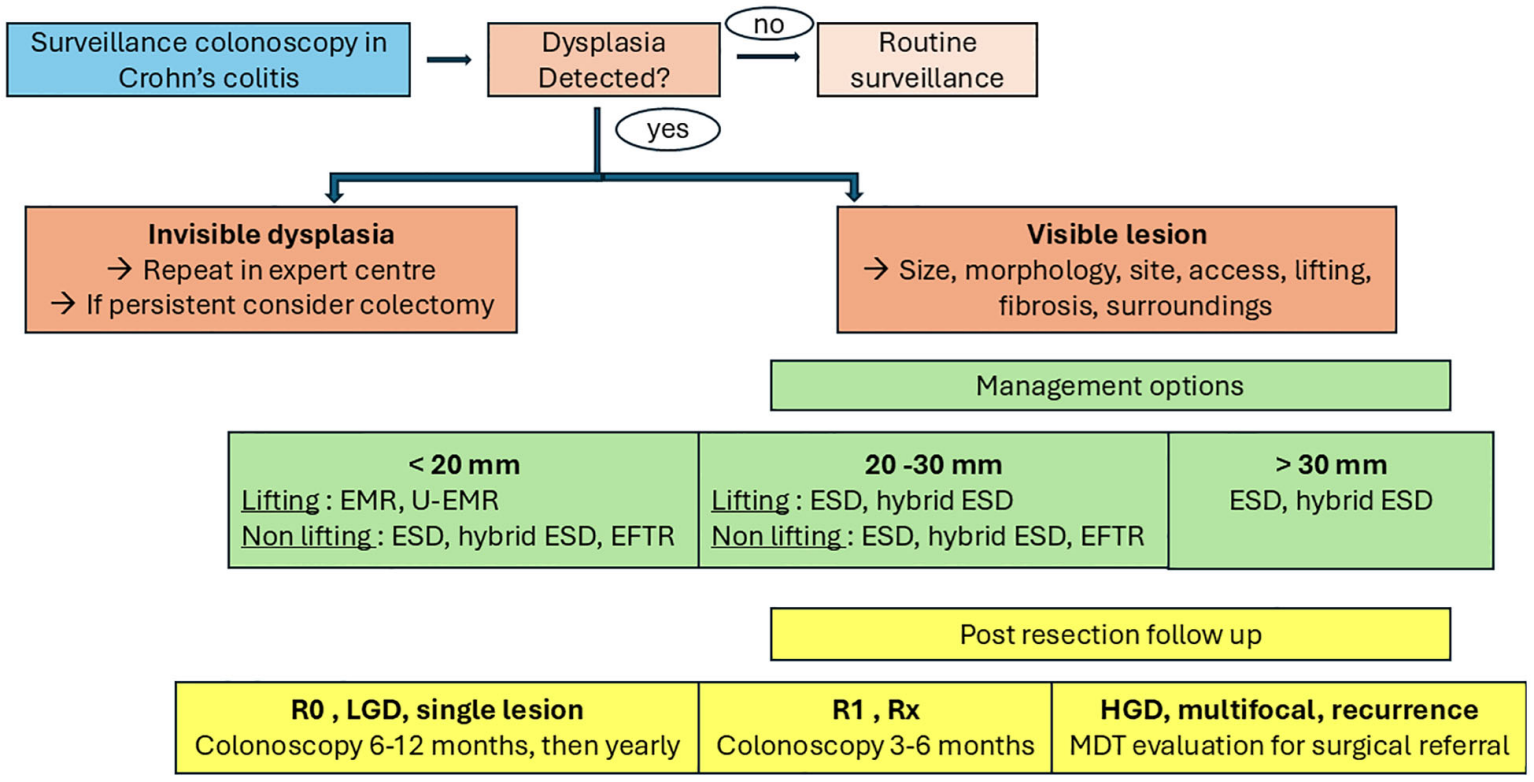
Flowchart outlining the endoscopic management of dysplasia in Crohn’s colitis. EMR, Endoscopic Mucosal Resection; U-EMR, Underwater Endoscopic Mucosal Resection; ESD, Endoscopic Submucosal Dissection; EFTR, Endoscopic Full-Thickness Resection; HGD, High-Grade Dysplasia; MTD, Multidisciplinary Team.

In CD, invisible dysplasia—dysplasia detected histologically without an identifiable lesion during endoscopy—requires careful reassessment. Once confirmed by expert histopathological review, patients should undergo HD endoscopic reevaluation, preferably at referral centers, to exclude subtle or previously overlooked lesions. Invisible dysplasia often reflects limitations of the initial exam, such as suboptimal bowel preparation or insufficient mucosal visualization (2, 29, 45). If confirmed and persistent despite advanced imaging, invisible dysplasia is generally an indication for colectomy due to the inability to target such lesions for endoscopic resection (46) and the risk of metachronous neoplasia (47). High-quality surveillance colonoscopy can detect most clinically relevant visible dysplasia and facilitate lesion characterization based on morphology (“The 5 S”: shape, size, site, surface, and surrounding mucosa) (48). Once a lesion is identified, it is essential to:

- differentiate colitis-associated neoplasia (CAN) from sporadic adenomas;
- assess whether en bloc resection is feasible (46);
- weigh the benefits and risks of endoscopic vs. surgical management within a multidisciplinary team (MTD) including endoscopists, gastroenterologists, and colorectal surgeons (49). Management should be individualized and referred to endoscopists with expertise in IBD-related dysplasia due to specific technical challenges: large non-pedunculated morphology, fibrosis, distorted submucosal planes, and surrounding inflammation (37). Both endoscopic mucosal resection (EMR) and endoscopic submucosal dissection (ESD) are valid approaches depending on lesion characteristics. A multicenter retrospective study evaluating distal cap-assisted EMR for adherent dysplastic lesions in IBD (12.5% CD) reported complete resection in 75% of cases, with no serious adverse events at 30-day follow-up (50). Underwater EMR (U-EMR) is a viable alternative for large, flat, or poorly lifting lesions, where submucosal fibrosis may limit conventional EMR. In a prospective study in UC patients, U-EMR achieved high en bloc and complete resection rates for lesions >20 mm, without increased perforation or post-polypectomy syndrome (51). ESD enables en bloc resection of larger non-polypoid lesions and may be preferred for non-lifting or superficially invasive lesions. A recent multicenter study of ESD and hybrid ESD for high-risk CAN reported an overall R0 resection rate of 85.4%. Outcomes were less favorable in CD than in UC (R0: 68.8% vs. 88.8%; adverse events: 25% vs. 10%) (52), highlighting the technical difficulties in CD linked to CD being a transmural disease with more submucosal fibrosis, leading to a higher rate of non-lifting polyps (52). A meta-analysis of 12 studies comprising 291 lesions showed en bloc resection in 92.5%, R0 in 81.5%, but a curative resection rate of only 48.9%, possibly due to advanced lesions (53). In contrast, a recent retrospective study of 82 IBD patients (19 with CD) reported a curative resection rate of 80% (54). Endoscopic full-thickness resection (EFTR), which enables transmural excision using an over-the-scope clip system, has emerged as an option for non-lifting or previously treated lesions in fibrotic or post-surgical colons. Though robust data are lacking, a recent case series demonstrated optimal R0 rates in anatomically challenging locations (55). EFTR is best suited for lesions ≤25–30 mm when other approaches are not feasible. Surgical resection remains the treatment of choice when endoscopic resection is incomplete, not technically feasible, or when histology shows high-risk features such as high-grade dysplasia or multifocality. According to recent BSG guidelines, patients undergoing endoscopic resection should receive a high-quality colonoscopy follow-up at 3–6 months, except for en bloc resection of <2 cm polypoid lesions with low-grade dysplasia, which may be followed up at 12 months. In cases of multifocal dysplasia, unresectable lesions, or multiple CRC risk factors, colectomy should be favored over continued surveillance (38) (**Figure 1**).

## 3 Crohn’s disease and small bowel cancer

### 3.1 Epidemiology and risk factors

Small bowel cancer (SBC) is a rare malignancy, accounting for less than 5% of all GI cancers. Patients with CD are at increased risk of small bowel adenocarcinoma (SBA) (4), as well as neuroendocrine neoplasms (NENs) and lymphoma. SBA is the most common form, with a 28-fold increased risk in CD and a poor prognosis (56). Despite this high relative risk, the absolute risk remains low—1.15 per 1,000 patients—with the ileum being the most frequently involved site (4, 57). Risk factors for SBC in CD include long disease duration, male sex, ileal or distal jejunal disease, strictures, penetrating disease, bypass loops, prior resections, and use of corticosteroids or immunomodulators (58). Conversely, small-bowel resection and aminosalicylate use appear protective (59). SBA has been associated with prior or synchronous ileal dysplasia, suggesting a dysplasia–adenocarcinoma sequence similar to the colon (60). Diagnosis is difficult; only 11% of cases show clear radiological signs, and symptoms often mimic a CD flare. Fewer than 5% of SBA cases are diagnosed preoperatively (61). For NENs, a population-based cohort study in Norway and Sweden of 142,008 IBD patients (10-year median follow-up) reported a 2.5-fold increased risk, especially in long-standing, stricturing or penetrating ileal disease (58, 61). A case-control study estimated the odds ratio (OR) for carcinoid tumors in CD at 14.9 (62). Unlike SBA, NENs are typically indolent, have favorable prognoses, and are often discovered incidentally (63). The incidence of small bowel lymphoma is also elevated in CD, with a 1.4–2-fold increase over the general population, though absolute risk is low (~0.26% over 10 years) (64). Literature remains inconclusive on whether the increased risk is due to immunosuppressants or IBD itself (65).

### 3.2 Endoscopy for detection of small bowel neoplasia: from small bowel capsule endoscopy and device assisted enteroscopy to future perspectives

No standardized surveillance protocols exist for SBC in CD. First, longitudinal studies assessing small bowel dysplasia and its progression to SBA are lacking. Second, the reduced accessibility of the small bowel and overlapping symptoms with inflammation make early diagnosis challenging. European guidelines recommend small bowel capsule endoscopy (SBCE) in patients at high risk for small-bowel tumors (66), such as those with unexplained iron-deficiency anemia or suspected metastases of unknown primary origin. Nonetheless, most SBCs are found during evaluation for obscure GI bleeding or anemia, and SBC is the cause in only 3.5–5% of these cases (67). In this context, SBCE shows superior diagnostic yield compared to push enteroscopy (68). Thus, ESGE guidelines—despite low evidence quality—recommend proceeding directly to SBCE unless there is risk of capsule retention (66). Balloon-assisted enteroscopy (BAE) allows deep small bowel access for diagnostic and therapeutic procedures, including biopsies and tattooing, with high rates of full small bowel examination using both oral and anal approaches (69). BAE is especially useful when imaging or SBCE identifies suspicious lesions, or for retrieving capsules and sampling inaccessible lesions (70–72). For primary surveillance, diagnostic performance data are limited due to low SBC incidence. However, in high-risk patients, SBCE or BAE may be considered. In a multicenter prospective study (73), 101 patients with long-standing ileal or jejunal CD underwent periodic upper/lower enteroscopy with DCE-guided and random biopsies. At one year, the prevalence of dysplasia or SBA was 4%, but the sensitivity of endoscopy for SBA was only 33%. Though rarely, small bowel dysplasia can be detected endoscopically (74), but current methods are inadequate for routine screening. Since SBC is often diagnosed intraoperatively in patients with long-standing stricturing CD (75), early surgical consideration may be more appropriate than prolonged medical management in this subgroup.

## 4 Discussion

Patients with Crohn’s colitis are at increased risk of CRC, although the risk varies substantially based on patient and disease characteristics. Despite confirmation by several meta-analyses, much of the available evidence derives from UC cohorts, limiting applicability to CD. As such, current prevention and surveillance strategies for CD are largely extrapolated from UC data, without adequately addressing CD’s distinct clinical, anatomical, and inflammatory features. GI endoscopy remains central for early dysplasia and CRC detection, but most comparative studies on endoscopic techniques include few CD patients, limiting evidence strength. Still, HD-WLE or VEC can be recommended as first-line tools for surveillance colonoscopy in colonic CD. DCE may be advantageous in high-risk or active disease due to VEC’s lower performance in inflamed mucosa. In such cases, random biopsies may enhance diagnostic yield. Advanced resection techniques—EMR, ESD, EFTR—have shown promise for managing dysplasia, though outcomes in CD appear less favorable than in UC, likely due to inflammation and fibrosis. These procedures should be reserved for selected patients and performed in specialized centers to optimize outcomes. CD-associated small bowel involvement substantially increases the risk of SBA, NENs, and lymphoma. Despite widespread use of imaging, SBCE, and BAE, early tumor diagnosis remains difficult. Current evidence does not support routine SBC surveillance, though high clinical suspicion is warranted with persistent or atypical symptoms. Early surgical evaluation should be considered in such contexts. Further prospective studies are urgently needed to guide individualized cancer prevention strategies in CD.
