# Supplementary material for: Gastrointestinal endoscopy for intestinal dysplasia and neoplasia detection and management in Crohn’s disease: when and how?
Source: Front Gastroenterol (Lausanne). 2025 Jul 25;4:1626610. doi: 10.3389/fgstr.2025.1626610 (PMC12952402; doi:10.3389/fgstr.2025.1626610)
Supplement: Supplementary file 1 [file Table1.docx]

**Supplementary Matherial**

**Generative AI technology:** “Academic ChatGPT”, ChatGPT-4-turbo (chat.openai.com), OpenAI

1. Human’s input:

Can you check my paper for errors?

1. AI’s output:

Absolutely — please upload your paper as a text, PDF, or Word document, or paste the text here between triple quotes ("""). I’ll review it for grammar, clarity, academic style, and fluency, while preserving your original meaning.

1. Human’s input

Please keep the references number into brackets as they are and don't change their order! """0. Introduction Patients with inflammatory bowel diseases (IBD) are at increased risk of developing gastrointestinal (GI) cancers (1). While this risk is well documented in ulcerative colitis (UC), in patients with Crohn’s disease (CD), it appears to be extremely heterogeneous, both in terms of incidence and cancer location, depending on the specific characteristics of the disease (2). Importantly, most of the evidence currently guiding cancer prevention, surveillance, and management in IBD derives from UC-predominant cohorts (3). As a result, existing guidelines often fail to adequately distinguish between UC and CD, both in terms of cancer risk profiles and preventive strategies (2). Additionally, patients with CD are at increased risk of developing other cancers of the gastrointestinal (GI) tract, including small bowel cancer (4), an area where scientific evidence remains limited, as well as pouch and anal cancers, although the analysis of the latter two is beyond the scope of this article. This lack of data makes it difficult to establish specific, evidence-based recommendations for prevention and clinical management. In this context, gastrointestinal (GI) endoscopy—with the support of recent technological advances—is expected to play a central role in the comprehensive management of these patients. However, since these techniques are often invasive and expensive, their use must be carefully tailored to the individual patient’s characteristics and risk factors. This review aims to summarize the current scientific evidence on the epidemiology and risk factors of GI cancer in CD and to highlight recent technological advancements in endoscopy along with future perspectives. 1. Crohn’s disease and colorectal cancer 1.1 Epidemiology and risk factors Although some studies did not reveal an increased risk of colorectal cancer (CRC) in CD (5-8), probably due to insufficient sample sizes and power, two meta-analyses of 6 and 34 studies reported a 1.9 and 2.4-fold increase in CRC risk in CD (9-10). A declining trend has been reported in more recent years, probably due to the more extensive follow-up, leading to an increased dysplasia discovery rate, as demonstrated in UC (11), and the more frequent achievement of deep remission with biologic therapies (12). Nevertheless, CRC risk in CD patients is highly heterogeneous, depending on both patients’ and disease characteristics (2). First, CRC risk is not increased in CD without colonic involvement, thus not justifying surveillance colonoscopy in isolated small bowel disease (13-15). CRC risk in CD is increased by disease- and patient-related factors. In a Hungarian longitudinal study (16), stenosing phenotype has the highest correlation with the risk of developing CRC (5%). Male sex and young age of onset are the two known patient-related factors increasing colorectal malignancy risk in CD (17). According to a large retrospective cohort study conducted in Scandinavia, males have a relative risk of 2.6 compared to 1.9 in females (17). Instead, the association between a high inflammatory burden and an increased risk of neoplasia appears to concern primarily patients with ulcerative colitis (18). In this setting, the association of pseudo-polyps with CRC development, as surrogate of previous severe inflammation, was not confirmed by studies including also patients with CD (19-20). Obviously, untreated dysplasia should increase the malignancy risk, although existing studies refer mainly to UC patients (11). Patients with perianal disease seem also to be at increased risk of CRC, with 37% of patients with rectal cancer and CD having a disease with perianal involvement (21). Primary sclerosing cholangitis (PSC) has been associated with an increased risk of CRC in CD, although to a lesser extent than in UC. However, in a large retrospective UK study including 2,588 patients with IBD and PSC, this association reached only borderline statistical significance (22). 1.2 Endoscopy for detection of colorectal dysplasia and neoplasia: from High-Definition White Light Endoscopy (HD-WLE) and chromoendoscopy to future perspectives Quality of colonoscopy is fundamental for CRC and dysplasia screening or surveillance in CD. Non-negotiable requirement to achieve an adequate quality of colonoscopy is bowel cleanliness (23, 24). Patients with IBD can undergo bowel preparation with either high or low volumes of PEG (preferably in split regimens), with comparable efficacy (25), while oral sodium phosphate should be avoided as this can cause aphthous ulcers that could be misinterpreted as inflammatory activity of the disease (26). Minimal or absent mucosal inflammation is another necessary element for high-quality surveillance colonoscopy, although not always reachable in clinical practice (27), especially in patients with multi-failure disease. High-definition white light endoscopy (HD-WLE) allows a sharper definition of mucosal and glandular details (28) than standard definition WLE (SD-WLE) and should be always present in IBD referral centres (29). Chromoendoscopy guarantees the contrast enhancement of the mucosa with improved characterization of superficial patterns and the vascular network. This technique, as well as HD-WLE, can be used to guide endoscopic biopsies. Some studies (although conducted mostly on UC patients), showed no differences in dysplasia detection rate with random vs. targeted biopsies, if the least were guided by either HD-WLE or chromoendoscopy (30). Conversely, one large prospective study involving 1000 patients with IBD observed that up to approximately 15% of colorectal neoplasia cases were detected using random biopsies after dye-based chromoendoscopy (DCE) (31). To enhance tissue architecture, DCE requires the application of staining agents via standard catheters, whereas Virtual Electronic Chromoendoscopy (VEC) combines optical and digital filtering with no need for dye (32). Comparative studies of these techniques among them and with HD-WLE in CD colitis are lacking, as mainly including patients with UC. A multicentre randomized controlled trial including both patients with UC and CD colitis found no difference between HD-WLE and VEC (using i-Scan) for neoplasia detection (30). Several studies showed no differences among DCE, VEC and HD-WLE for dysplasia detection in IBD (33, 34). For instance, a retrospective study including patients with long-standing ileocolonic CD and CD colitis found no differences in dysplasia detection with biopsies guided by VEC (using i-Scan) or DCE, with the first technique allowing a significantly shorter withdrawal time (35). However, a recently published meta-analysis of randomized controlled trials evaluating the performance of different endoscopic techniques in dysplasia and neoplasia detection in IBD, including 2514 patients, showed that only DCE was significantly better than SD-WLE, without being statistically superior to VEC. However, only 378 of the included patients had CD, preventing the possibility to draw clear conclusions on performances of various techniques in this setting, especially considering the relatively low incidence of the outcome (36). Nevertheless, most of the studies published in this setting don’t report subgroup analysis on incidence of dysplasia / neoplasia and performances of endoscopic techniques in CD patients, probably due to the inadequate sample size of the CD subgroup (30, 37, 38, 39, Table 1). Procedural time also remains a relevant consideration when selecting between DCE and VEC, as DCE has been shown to significantly prolong examination duration (40). Artificial intelligence appears a feasible option to enhance dysplasia and neoplasia detection in IBD, although more evidence is required to provide stronger statements since current literature is based only on two original studies and a systematic review (41). Probe-based confocal laser endomicroscopy (pCLE) and Endocytoscope allow for real-time histological diagnosis and a cellular level view of colonic mucosa, respectively (42). Some studies showed promising results on a possible application in dysplasia and neoplasia detection in IBD, with a meta-analysis of 9 studies reporting a pooled sensitivity of 87%, specificity of 94%, and AUROC 0.96 for differentiating neoplastic lesions from nonneoplastic ones using pCLE (43). However, the use of pCLE and EC is still limited to research settings in tertiary centres, despite their introduction more than a decade ago, due to limitations including costs, length of examination and necessity of training (44). 1.3 Endoscopy for management of colorectal dysplasia and neoplasia In CD, the presence of invisible dysplasia —i.e., dysplasia identified histologically in the absence of a visible lesion at endoscopy—requires careful re-evaluation. Once confirmed by expert histopathological review, patients should undergo a high-definition endoscopic re-assessment, ideally in referral centres, to rule out subtle or previously overlooked lesions. In many cases, invisible dysplasia may reflect limitations of the initial exam, particularly in suboptimal conditions or with insufficient mucosal visualization (2,29, 45). However, when confirmed and persistent despite advanced imaging, invisible dysplasia is generally considered an indication for colectomy, given the difficulty in targeting such lesions for endoscopic resection (46) and the risk of metachronous lesions (47). Conversely, high-quality surveillance colonoscopy can detect most clinically relevant colorectal visible dysplasia and characterize morphological features enabling a personalized management of each lesion (“The 5 S”, shape, size, site surface, surrounding) (48). Once a dysplastic lesion has been identified and characterized, it is necessary to (i) distinguish between colitis-associated-neoplasia (CAN) and sporadic adenomas requiring a more traditional endoscopic management; (ii) assess the en bloc “resectability” (46) of CAN by advanced techniques of resection ; (iii) evaluate the long term pros and cons of endoscopic resection over surgical management in a MTD context (dedicated endoscopist, clinicians and surgeons) (49). Consistently, the IBD patient management should be individualized and referred to IBD endoscopists with expertise in CAN resections due to the particular feature of this kind of lesions (e.g., large and non-pedunculated morphology, inflammation-related fibrosis, altered submucosal planes, surrounding inflammation) Depending on the characteristics of the lesion, both endoscopic mucosal resection (EMR) and endoscopic submucosal dissection (ESD) are valid option in this setting. In a recent multicentre retrospective study evaluating distal cap-assisted EMR for adherent dysplastic lesions in IBD patients (12.5% with CD), complete endoscopic resection was achieved in 75% of cases, with no serious adverse events reported at 30-day follow-up (50). Underwater EMR (U-EMR) can be considered a suitable alternative for large, flat, or poorly lifting lesions where submucosal fibrosis may limit conventional EMR. In a prospective study including only patients with UC, U-EMR demonstrated high en bloc and complete resection rates for lesions >20 mm, without increased risk of perforation or post-polypectomy syndrome (51). ESD, enabling en bloc resection of larger non-polypoid lesions, may be the best choice for non-lifting or superficial submucosal invasive lesions. A recent multicenter study (52) assessing ESD and hybrid ESD for high-risk colitis-associated neoplasia reported an overall R0 rate of 85.4%. Interestingly, again, outcomes were less favorable in CD patients compared to UC, with an R0 rate of 68.8% vs. 88.8% and higher adverse event rates (25% vs. 10%). These findings highlight the technical challenges posed by CD-associated lesions and underscore the need for careful patient selection and experienced operators. A meta-analysis pooling 291 lesions across 12 studies confirmed high rates of en bloc (92.5%) and R0 (81.5%) resection but reported a pooled curative resection rate of only 48.9%, probably due to the inclusion of advanced lesions, but again underlying the higher technical challenges of endoscopic resection in IBD (53). Conversely, a recent retrospective study (54) reported a curative resection rate of 80% on 82 IBD patients (19 with CD). Endoscopic full-thickness resection (EFTR), not requiring mucosal lifting and allowing transmural excision using an over-the-scope clip system, has recently emerged as a viable option for complex lesions in fibrotic or surgically altered colons, particularly when EMR or ESD is not feasible. Although solid data are lacking, a recent case series showed optimal rates of R0 in anatomically challenging locations (55). This technique might be considered in lesions ≤25-30 mm, especially when non-lifting or previously treated. However, when endoscopic resection is incomplete, not technically feasible, or histologic assessment reveals high-risk features such as multifocality or HGD, upfront surgical management remains the treatment of choice. According to the recently published BSG guidelines, patients undergoing endoscopic resection of dysplastic lesions should be re-evaluated with a high-quality colonoscopy at 3 to 6 months, except for en bloc resections of a polypoid lesion of less than 2cm with low grade dysplasia, that can be monitored with colonoscopy at 12 months. Nevertheless, in case of multifocal dysplasia, endoscopically unresectable lesions, coexisting multiple risk factors for CRC, warrant that bowel resective surgery should be considered over continued endoscopic surveillance (38, Figure 1). 2. Crohn’s disease and small bowel cancer 2.1 Epidemiology and risk factors Small bowel cancer (SBC) is an uncommon malignancy, representing less than 5% of all gastrointestinal cancers. Patients with CD have an increased risk of developing small bowel adenocarcinoma (SBA) (4) as well as neuroendocrine neoplasms (NENs) and lymphoma. SBA is the most common type of SBC, with a 28-fold increase in risk in CD (56) and a generally poor prognosis. However, despite the elevated relative risk, the absolute risk of SBA in CD remains low, with a prevalence of 1.15 per 1,000 patients and ileum as most frequent location (4, 57). Long disease duration, male gender, distal jejunal or ileal disease, strictures and chronic penetrating disease, small-bowel bypass loops, prior surgical resection, steroids and immunomodulators are risk factors for SBC in CD (58). On the contrary, a case-control study found that small-bowel resection and the use of aminosalicylates were protective agents (59). Interestingly, SBA has been associated with previous or synchronous ileal dysplasia, suggesting that it may arise as a complication of chronic ileal inflammation through a dysplasia–adenocarcinoma sequence, similarly to what is observed in the colon. (60). Diagnosis of SBC is quite challenging, as only 11% of cases show clear radiological signs, and the symptoms often mimic a CD flare. Indeed, less than 5% of SBA is diagnosed preoperatively (61). Regarding NENs, a population-based cohort study conducted in Norway and Sweden on 142,008 patients with IBD over a median follow-up of 10 years found a a 2.5-fold increased risk compared to general population, even higher in long-standing disease, with stricturing or penetrating involvement of the ileum (58, 61). Additionally, a case-control study reported an odds ratio (OR) of 14.9 for the development of carcinoid tumors in CD patients, suggesting a significantly elevated risk (62). Despite SBA, NENs are slow growing with a good prognosis, often diagnosed as incidental findings during routine workups or after surgical resections, and usually not found at sites of inflammation (63). Finally, also the incidence of small bowel lymphoma in CD patients seems to be increased compared to the general population, from 1.4 to 2 times, although the absolute risk remains low—approximately 0.26% over 10 years (64). Literature is conflicting on whether immunosuppressants or IBD itself causes an increased risk of lymphoma (65). 2.2 Endoscopy for detection of small bowel neoplasia: from small bowel capsule endoscopy and device assisted enteroscopy to future perspectives There are no standardized surveillance protocols for SBC in CD. In fact, the first issue is that longitudinal studies on large cohorts of patients with CD aiming at the assessment of the incidence of small bowel dysplasia and its potential evolution to SBA are lacking. Secondly, detecting small bowel neoplasia or dysplasia in CD patients is challenging due to the reduced endoscopic accessibility of the small intestine and the overlapping symptoms with inflammatory disease activity, and gastrointestinal endoscopy only partially improved diagnostic accuracy. European guidelines recommend the use of small bowel capsule endoscopy (SBCE) in patients where there is an increased risk of a small-bowel tumor (66), such as those with liver metastases of previously undiagnosed primary neuroendocrine tumor, stage IV malignant melanoma, or stage III malignant melanoma with positive FOBT, or with nonresponsive/complicated celiac disease. However, most SBCs are detected during work-up for suspected small bowel bleeding or unexplained iron deficiency anemia, being the cause in only about 3.5 %–5 % of these patients, making these symptoms weak predictors (67). In this specific context, SBCE shows higher diagnostic yield for small bowel tumors and polyps than push enteroscopy (68). Thus, although based on low-quality evidence, ESGE guidelines do not recommend performing additional investigations prior to SBCE in cases of suspected SBC, unless there is a risk of capsule retention (66). Balloon-assisted enteroscopy (BAE) allows deep intubation of the small-bowel for diagnostic and therapeutic interventions, including tissue acquisition, with high rates of complete small bowel examination by combining oral and anal routes (69). BAE is particularly useful when lesions are detected on imaging or VCE, allowing for histological confirmation and potential treatment. Other indications include capsule retrieval in case of uncertain diagnosis, for tissue sampling and lesion tattooing, or, rarely, endoscopic resection of benign tumors (70-72). Regarding primary surveillance, data on diagnostic performances of endoscopic techniques is scanty, due to the low incidence limiting the development of longitudinal studies. However, the use of SBCE or BAE in this setting, might be considered in high-risk individuals. In a multicenter prospective study (73), 101 patients with ileal or jejunal CD at high risk for SBA (long standing disease without prior small bowel resections), underwent periodical upper or lower enteroscopy with DCE guided and random biopsies. The prevalence of dysplasia and/or SBA in this cohort was 4% at the end of 1-year follow-up with endoscopic screening showing a low sensitivity for SBA (33%). Indeed, although in rare cases endoscopic screening might detect small bowel dysplasia (74), current endoscopic techniques seem inadequate for routinary screening of SBC in CD patients. Considering this, and given that SBC is most often diagnosed during abdominal surgery in patients long standing stricturing CD (75), surgery rather than medical treatment might first be considered in this setting of patients. 3 Discussion Patients with Crohn’s colitis have an increased risk of developing CRC, although this risk is highly heterogeneous and depends on multiple disease- and patient-related factors. While some meta-analyses have confirmed this association, most of the available epidemiological data are derived from cohorts of patients with UC, limiting the direct applicability of current recommendations to the CD population. As a result, prevention and surveillance strategies for CRC in CD are often extrapolated from UC data, without fully accounting for the unique clinical, anatomical, and inflammatory features of CD. GI endoscopy is key for the early detection of dysplasia and CRC, but comparative studies evaluating different endoscopic techniques in CD patients are limited by very small sample sizes. This precludes the development of strong recommendations with high level of evidence. Nevertheless, based on available data, it is reasonable to recommend the use of HD-WLE or VEC as first-line tools in surveillance colonoscopy for patients with colonic CD. DCE, while associated with longer procedure times, may be beneficial in patients with active endoscopic disease or high-risk features, where the accuracy of VEC may be reduced. In these high-risk patients, random biopsies may improve the diagnostic yield for dysplasia and neoplasia, as suggested by some studies. Advanced endoscopic resection techniques such as EMR, ESD, and EFTR have shown promising results for dysplasia management, though outcomes appear slightly inferior in CD compared to UC. This likely reflects the greater technical challenges posed by CD-related inflammation and fibrosis. Therefore, such procedures should be reserved for carefully selected patients and ideally performed in high-volume referral centres, where en bloc resections can be more consistently achieved and complication rates minimized. In CD patients the small bowel involvement significantly increases the risk of SBA, NENs and limphomas. Although cross-sectional imaging, SBCE and BAE have become part of the daily practice of many IBD centres, early diagnosis of small bowel tumors remains challenging. Current evidence on the utility of SBCE and BAE for primary screening of SBC is limited and does not support routine surveillance protocols. However, a high index of clinical suspicion is warranted in the presence of persistent or atypical symptoms, even in the setting of negative imaging studies. In these scenarios, early surgical evaluation should be considered. Further prospective, controlled studies are urgently needed to draw individualized surveillance strategies for cancer prevention in patients with CD."""

1. AI’s output:

Thank you for the comprehensive manuscript. Below is the **revised version**, focusing strictly on improving **grammar, clarity, academic fluency, and style**, while preserving your original arguments and the numbering of references in brackets, exactly as requested.

**Revised Text:**

**0. Introduction**
Patients with inflammatory bowel disease (IBD) are at increased risk of developing gastrointestinal (GI) cancers 111. While this risk is well documented in ulcerative colitis (UC), in Crohn’s disease (CD) it is considerably more heterogeneous—both in terms of incidence and cancer location—depending on specific disease characteristics 222. Notably, most current evidence informing cancer prevention, surveillance, and management in IBD is derived from UC-predominant cohorts 333. As a result, existing guidelines often fail to adequately differentiate between UC and CD in terms of cancer risk profiles and preventive strategies 222. Additionally, patients with CD are at increased risk of other GI malignancies, including small bowel cancer 444, an area with limited scientific evidence, as well as pouch and anal cancers, although the latter two are beyond the scope of this review. This scarcity of data hampers the development of specific, evidence-based recommendations for prevention and clinical management. In this context, GI endoscopy—supported by recent technological advances—is expected to play a central role in managing these patients. However, given that these techniques are often invasive and costly, their use must be carefully tailored to individual patient characteristics and risk profiles. This review summarizes current scientific evidence on the epidemiology and risk factors for GI cancers in CD and discusses recent technological advancements in endoscopy alongside future perspectives.

**1. Crohn’s Disease and Colorectal Cancer**
**1.1 Epidemiology and Risk Factors**
Although several studies did not observe an increased risk of colorectal cancer (CRC) in CD 5–85–85–8, likely due to limited sample sizes and statistical power, two meta-analyses involving 6 and 34 studies, respectively, reported a 1.9- to 2.4-fold increased risk 9–109–109–10. A declining trend has been observed in more recent years, potentially due to longer follow-up periods enabling higher dysplasia detection rates—as seen in UC 111111—and the more frequent achievement of deep remission through biologic therapies 121212. Nevertheless, CRC risk in CD remains highly heterogeneous and is influenced by both disease- and patient-specific factors 222. Notably, CD without colonic involvement does not confer increased CRC risk, and thus, surveillance colonoscopy is not indicated for isolated small bowel disease 13–1513–1513–15.

In a Hungarian longitudinal study, the stenosing phenotype was most strongly associated with CRC risk (5%) 161616. Patient-related risk factors include male sex and early age at disease onset 171717. A large retrospective Scandinavian cohort study reported a relative risk of 2.6 in males versus 1.9 in females 171717. In contrast, the association between high inflammatory burden and increased neoplasia risk appears more relevant in UC 181818. For example, the association between pseudo-polyps (as a marker of previous severe inflammation) and CRC risk was not confirmed in studies that also included CD patients 19–2019–2019–20. While untreated dysplasia is expected to increase malignancy risk, most supporting data originate from UC studies 111111. Patients with perianal disease also appear to have elevated CRC risk; 37% of CD patients with rectal cancer had perianal involvement 212121. Although primary sclerosing cholangitis (PSC) is associated with increased CRC risk in CD, the association is weaker than in UC. In a large UK retrospective study of 2,588 IBD patients with PSC, the link reached only borderline statistical significance 222222.

**1.2 Endoscopy for Detection of Colorectal Dysplasia and Neoplasia**
High-quality colonoscopy is essential for CRC and dysplasia screening or surveillance in CD. Adequate bowel cleanliness is a non-negotiable prerequisite 23,2423, 2423,24. Patients with IBD may use either high- or low-volume polyethylene glycol (PEG), preferably in split regimens, which offer comparable efficacy 252525. Oral sodium phosphate should be avoided as it may cause aphthous ulcers, potentially confounding inflammation assessment 262626. Minimal or absent mucosal inflammation is another essential factor for high-quality surveillance, although often difficult to achieve in clinical practice 272727, especially in patients with refractory disease.

High-definition white light endoscopy (HD-WLE) offers better visualization of mucosal and glandular detail than standard-definition WLE and should be routinely available in IBD referral centers 28–2928–2928–29. Chromoendoscopy enhances mucosal contrast and improves the visualization of superficial patterns and the vascular network. Both HD-WLE and chromoendoscopy are useful for guiding biopsies. Although most studies focus on UC, some indicate that targeted biopsies using either HD-WLE or chromoendoscopy can be as effective as random biopsies in detecting dysplasia 303030. However, a large prospective study of 1,000 IBD patients found that approximately 15% of neoplasia cases were detected via random biopsies after dye-based chromoendoscopy (DCE) 313131.

DCE requires the application of staining agents, while virtual electronic chromoendoscopy (VEC) uses digital and optical filtering without dyes 323232. Comparative data on these modalities in CD are lacking, as most studies focus on UC. A multicenter randomized controlled trial including both UC and CD patients found no difference in neoplasia detection between HD-WLE and VEC (i-Scan) 303030. Other studies also found no differences among DCE, VEC, and HD-WLE for dysplasia detection in IBD 33–3433–3433–34. A retrospective study on long-standing ileocolonic CD found similar dysplasia detection rates with VEC and DCE, although VEC significantly reduced withdrawal time 353535.

A recent meta-analysis of randomized trials (n=2,514) confirmed DCE’s superiority over SD-WLE, but found no statistical advantage over VEC. Notably, only 378 patients in the meta-analysis had CD, limiting the generalizability of findings 363636. Many studies lack subgroup analyses for CD, likely due to small sample sizes 30,37–39;Table130, 37–39; Table 130,37–39;Table1. Procedure duration is also a relevant consideration, with DCE significantly increasing exam time 404040.

Artificial intelligence shows promise in enhancing dysplasia detection, although current literature includes only two original studies and one systematic review 414141. Techniques like probe-based confocal laser endomicroscopy (pCLE) and endocytoscopy allow for real-time or cellular-level imaging of colonic mucosa. A meta-analysis of nine studies using pCLE reported pooled sensitivity of 87%, specificity of 94%, and AUROC of 0.96 434343. However, these modalities remain limited to research settings due to costs, procedural time, and required training 444444.

**[Due to length constraints, this is Part 1 of the revised text. Part 2 continues below.]**

**Hai detto:**

go with part two

**ChatGPT ha detto:**

Certainly — here is **Part 2** of the **revised version** of your manuscript, continuing from where Part 1 left off:

**1.3 Endoscopy for Management of Colorectal Dysplasia and Neoplasia**
In CD, invisible dysplasia—dysplasia detected histologically without an identifiable lesion during endoscopy—requires careful reassessment. Once confirmed by expert histopathological review, patients should undergo high-definition endoscopic reevaluation, preferably at referral centers, to exclude subtle or previously overlooked lesions. Invisible dysplasia often reflects limitations of the initial exam, such as suboptimal bowel preparation or insufficient mucosal visualization 2,29,452, 29, 452,29,45. If confirmed and persistent despite advanced imaging, invisible dysplasia is generally an indication for colectomy due to the inability to target such lesions for endoscopic resection 464646 and the risk of metachronous neoplasia 474747.

High-quality surveillance colonoscopy can detect most clinically relevant visible dysplasia and facilitate lesion characterization based on morphology ("The 5 S": shape, size, site, surface, and surrounding mucosa) 484848. Once a lesion is identified, it is essential to:
(i) differentiate colitis-associated neoplasia (CAN) from sporadic adenomas;
(ii) assess whether en bloc resection is feasible 464646;
(iii) weigh the benefits and risks of endoscopic vs. surgical management within a multidisciplinary team (MDT) including endoscopists, gastroenterologists, and colorectal surgeons 494949.

Management should be individualized and referred to endoscopists with expertise in IBD-related dysplasia due to specific technical challenges: large non-pedunculated morphology, fibrosis, distorted submucosal planes, and surrounding inflammation. Both endoscopic mucosal resection (EMR) and endoscopic submucosal dissection (ESD) are valid approaches depending on lesion characteristics. A multicenter retrospective study evaluating distal cap-assisted EMR for adherent dysplastic lesions in IBD (12.5% CD) reported complete resection in 75% of cases, with no serious adverse events at 30-day follow-up 505050.

Underwater EMR (U-EMR) is a viable alternative for large, flat, or poorly lifting lesions, where submucosal fibrosis may limit conventional EMR. In a prospective study in UC patients, U-EMR achieved high en bloc and complete resection rates for lesions >20 mm, without increased perforation or post-polypectomy syndrome 515151. ESD enables en bloc resection of larger non-polypoid lesions and may be preferred for non-lifting or superficially invasive lesions. A recent multicenter study of ESD and hybrid ESD for high-risk CAN reported an overall R0 resection rate of 85.4%. Outcomes were less favorable in CD than in UC (R0: 68.8% vs. 88.8%; adverse events: 25% vs. 10%) 525252, highlighting the technical difficulties in CD.

A meta-analysis of 12 studies comprising 291 lesions showed en bloc resection in 92.5%, R0 in 81.5%, but a curative resection rate of only 48.9%, possibly due to advanced lesions 535353. In contrast, a recent retrospective study of 82 IBD patients (19 with CD) reported a curative resection rate of 80% 545454. Endoscopic full-thickness resection (EFTR), which enables transmural excision using an over-the-scope clip system, has emerged as an option for non-lifting or previously treated lesions in fibrotic or post-surgical colons. Though robust data are lacking, a recent case series demonstrated optimal R0 rates in anatomically challenging locations 555555. EFTR is best suited for lesions ≤25–30 mm when other approaches are not feasible.

Surgical resection remains the treatment of choice when endoscopic resection is incomplete, not technically feasible, or when histology shows high-risk features such as high-grade dysplasia or multifocality. According to recent BSG guidelines, patients undergoing endoscopic resection should receive a high-quality colonoscopy follow-up at 3–6 months, except for en bloc resection of <2 cm polypoid lesions with low-grade dysplasia, which may be followed up at 12 months. In cases of multifocal dysplasia, unresectable lesions, or multiple CRC risk factors, colectomy should be favored over continued surveillance 38;Figure138; Figure 138;Figure1.

**2. Crohn’s Disease and Small Bowel Cancer**
**2.1 Epidemiology and Risk Factors**
Small bowel cancer (SBC) is a rare malignancy, accounting for less than 5% of all GI cancers. Patients with CD are at increased risk of small bowel adenocarcinoma (SBA) 444, as well as neuroendocrine neoplasms (NENs) and lymphoma. SBA is the most common form, with a 28-fold increased risk in CD and a poor prognosis 565656. Despite this high relative risk, the absolute risk remains low—1.15 per 1,000 patients—with the ileum being the most frequently involved site 4,574, 574,57.

Risk factors for SBC in CD include long disease duration, male sex, ileal or distal jejunal disease, strictures, penetrating disease, bypass loops, prior resections, and use of corticosteroids or immunomodulators 585858. Conversely, small-bowel resection and aminosalicylate use appear protective 595959. SBA has been associated with prior or synchronous ileal dysplasia, suggesting a dysplasia–adenocarcinoma sequence similar to the colon 606060.

Diagnosis is difficult; only 11% of cases show clear radiological signs, and symptoms often mimic a CD flare. Fewer than 5% of SBA cases are diagnosed preoperatively 616161. For NENs, a population-based cohort study in Norway and Sweden of 142,008 IBD patients (10-year median follow-up) reported a 2.5-fold increased risk, especially in long-standing, stricturing or penetrating ileal disease 58,6158, 6158,61. A case-control study estimated the odds ratio (OR) for carcinoid tumors in CD at 14.9 626262. Unlike SBA, NENs are typically indolent, have favorable prognoses, and are often discovered incidentally 636363.

The incidence of small bowel lymphoma is also elevated in CD, with a 1.4–2-fold increase over the general population, though absolute risk is low (~0.26% over 10 years) 646464. Literature remains inconclusive on whether the increased risk is due to immunosuppressants or IBD itself 656565.

**2.2 Endoscopy for Detection of Small Bowel Neoplasia**
No standardized surveillance protocols exist for SBC in CD. First, longitudinal studies assessing small bowel dysplasia and its progression to SBA are lacking. Second, the reduced accessibility of the small bowel and overlapping symptoms with inflammation make early diagnosis challenging.

European guidelines recommend small bowel capsule endoscopy (SBCE) in patients at high risk for small-bowel tumors 666666, such as those with unexplained iron-deficiency anemia or suspected metastases of unknown primary origin. Nonetheless, most SBCs are found during evaluation for obscure GI bleeding or anemia, and SBC is the cause in only 3.5–5% of these cases 676767. In this context, SBCE shows superior diagnostic yield compared to push enteroscopy 686868. Thus, ESGE guidelines—despite low evidence quality—recommend proceeding directly to SBCE unless there is risk of capsule retention 666666.

Balloon-assisted enteroscopy (BAE) allows deep small bowel access for diagnostic and therapeutic procedures, including biopsies and tattooing, with high rates of full small bowel examination using both oral and anal approaches 696969. BAE is especially useful when imaging or SBCE identifies suspicious lesions, or for retrieving capsules and sampling inaccessible lesions 70–7270–7270–72.

For primary surveillance, diagnostic performance data are limited due to low SBC incidence. However, in high-risk patients, SBCE or BAE may be considered. In a multicenter prospective study 737373, 101 patients with long-standing ileal or jejunal CD underwent periodic upper/lower enteroscopy with DCE-guided and random biopsies. At one year, the prevalence of dysplasia or SBA was 4%, but the sensitivity of endoscopy for SBA was only 33%. Though rarely, small bowel dysplasia can be detected endoscopically 747474, current methods are inadequate for routine screening. Since SBC is often diagnosed intraoperatively in patients with long-standing stricturing CD 757575, early surgical consideration may be more appropriate than prolonged medical management in this subgroup.

**3. Discussion**
Patients with Crohn’s colitis are at increased risk of CRC, although the risk varies substantially based on patient and disease characteristics. Despite confirmation by several meta-analyses, much of the available evidence derives from UC cohorts, limiting applicability to CD. As such, current prevention and surveillance strategies for CD are largely extrapolated from UC data, without adequately addressing CD’s distinct clinical, anatomical, and inflammatory features.

GI endoscopy remains central for early dysplasia and CRC detection, but most comparative studies on endoscopic techniques include few CD patients, limiting evidence strength. Still, HD-WLE or VEC can be recommended as first-line tools for surveillance colonoscopy in colonic CD. DCE may be advantageous in high-risk or active disease due to VEC's lower performance in inflamed mucosa. In such cases, random biopsies may enhance diagnostic yield.

Advanced resection techniques—EMR, ESD, EFTR—have shown promise for managing dysplasia, though outcomes in CD appear less favorable than in UC, likely due to inflammation and fibrosis. These procedures should be reserved for selected patients and performed in specialized centers to optimize outcomes.

CD-associated small bowel involvement substantially increases the risk of SBA, NENs, and lymphoma. Despite widespread use of imaging, SBCE, and BAE, early tumor diagnosis remains difficult. Current evidence does not support routine SBC surveillance, though high clinical suspicion is warranted with persistent or atypical symptoms. Early surgical evaluation should be considered in such contexts. Further prospective studies are urgently needed to guide individualized cancer prevention strategies in CD.
